# Efficacy of a spot-on combination containing 10% w/v imidacloprid and 1% w/v moxidectin for the treatment of troglostrongylosis in experimentally infected cats

**DOI:** 10.1186/s13071-022-05185-y

**Published:** 2022-02-22

**Authors:** Donato Traversa, Katharina Raue, Hannah Ringeisen, Katrin Blazejak, Katrin Bisterfeld, Angela Di Cesare, Mariasole Colombo, Claudia Böhm, Christina Strube, Matthias Pollmeier

**Affiliations:** 1 Faculty of Veterinary Medicine, Teramo, Italy; 2 Institute for Parasitology, Centre for Infection Medicine, University of Veterinary Medicine Hannover, Hanover, Germany; 3 Elanco Animal Health, Bayer Animal Health GmbH, Monheim, Germany

**Keywords:** Cat, Moxidectin, Treatment, Feline lungworms, *Troglostrongylus brevior*

## Abstract

**Background:**

Parasitic bronchopneumonia in domestic cats in Europe, which can manifest with moderate to severe clinical signs, is frequently caused by *Troglostrongylus brevior*. Data on epizootiological and clinical relevance of cat troglostrongylosis have been published in the last decade but treatment options are still limited. Promising effectiveness data have been generated from clinical cases and field trials for a spot-on formulation containing 1% w/v moxidectin and 10% w/v imidacloprid (Advocate^®^, Elanco Animal Health). Therefore, two studies have been conducted to confirm under experimental conditions the efficacy of moxidectin 1% contained in Advocate^®^ for the treatment of cat troglostrongylosis.

**Methods:**

Sixteen and 20 cats experimentally infected with *T. brevior* were included in two separate studies, i.e., Study 1 and 2, respectively. Cats were infected with *T. brevior* third-stage larvae via gastric tube. In both studies cats were randomized to untreated (control, Group 1) and treatment (Group 2) groups. In Study 1 and Study 2, the two groups comprised eight and 10 cats each. Treated cats received Advocate^®^ spot-on twice at a 4-week interval. The primary efficacy criterion was the number of viable adult *T. brevior* counted at necropsy. Throughout the trial, the fecal shedding of first-stage larvae (L1) was assessed in treated and untreated control cats.

**Results:**

The experimental model was successful in both studies, as all cats started shedding *T. brevior* L1 within 25 days post-infection. At necropsy, *T. brevior* adults were found in 4/8 and 4/10 cats of the control groups in Study 1 and 2, respectively, while none of the treated cats harbored adult worms. The necropsy worm counts in controls did not meet relevant guideline requirements for adequacy of infection, with fewer than six infected cats in the control groups, thus limiting conclusions on treatment efficacy. The fact that 6/8 and 8/10 control cats in Study 1 and 2, respectively, shed L1 up to necropsy while larval shedding ceased in all treated animals after the first treatment provides supporting evidence on the level of efficacy. No remarkable adverse events were recorded in the two studies.

**Conclusion:**

These results indicate that Advocate^®^ spot-on is a safe and effective option for treating cats infected by *T. brevior*.

**Graphical Abstract:**

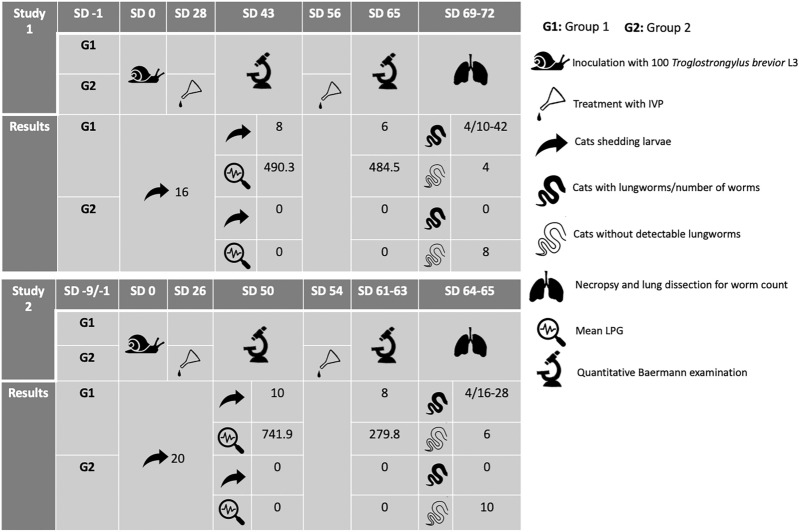

## Background

The cat lungworm *Aelurostrongylus abstrusus* has long been recognized as the only metastrongyloid nematode affecting the airways of domestic cats. However, in the last decade the closely related crenosomatid *Troglostrongylus brevior*, previously related only to wild felids, has been regarded as a primary cause of verminous bronchopneumonia in cat populations of Europe, mainly in countries of the Mediterranean basin [1, 2]. At the adult stage, *T. brevior* lives in the bronchi and bronchioles of the felid host and its biological cycle overlaps that of other gastropod-transmitted metastrongyloids, though it may also be transmitted vertically from the queen to the kittens, most probably via the milk [2, 3]. Horizontal infections occur via the ingestion of third-stage infective larvae (L3) harbored by terrestrial mollusks (intermediate hosts) or small prey (paratenic hosts) [2].

Several drivers, including a spill-over from the European wildcat (i.e., the natural host, *Felis silvestris*) and biological and climatic factors, have been suggested to explain an apparent spreading of troglostrongylosis [2, 4]. Today, in many regions of Europe, *T. brevior* is recognized as a major respiratory parasite of both domestic cats and wildcats, existing in sympatry with *A. abstrusus*, and in some territories troglostrongylosis is more prevalent than aelurostrongylosis in domestic cats [5–12].

Feline troglostrongylosis is characterized by catarrhal bronchitis and interstitial pneumonia, which are particularly severe and potentially life-threatening in kittens and young animals [2, 13]. Infected cats display ocular-nasal discharge, sneezing, dyspnea, tachypnea, cough, and non-specific clinical signs, e.g., hypo- or anorexia, hyperthermia, dehydration [1, 11, 14–17].

Despite the growing importance of troglostrongylosis in feline medicine, control options are still limited. Only two formulations containing eprinomectin are currently labelled in Europe for the treatment of troglostrongylosis but other molecules, including moxidectin, have shown promising efficacy against *T. brevior* [15, 16, 18–22]. Therefore, two in vivo studies were conducted to further investigate the efficacy of a spot-on combination containing 10% w/v imidacloprid and 1% w/v moxidectin (Advocate^®^ for cats, Elanco Animal Health) in the treatment of *T. brevior* infection in experimentally infected cats.

## Methods

### Study design

Two blinded, controlled, randomized, single-site efficacy studies were conducted in accordance with the International Cooperation on Harmonisation of Technical Requirements for Registration of Veterinary Medicinal Products (VICH) Guideline (GL) 7, “Efficacy of anthelmintics: general requirements”, VICH GL 9, “Guideline on Good Clinical Practice”, and VICH GL 20, “Efficacy of anthelmintics: specific recommendations for felines” [23–25]. The studies were carried out using cats as animal models, because there are no in vitro methods available to simulate actual in vivo models for this category of efficacy evaluations.

### Study animals

#### Acclimatization and housing

In both studies, the husbandry of the cats complied with the Directive 2010/63/EU of the European Parliament and of the council of 22 September 2010 on the protection of the animals used for scientific purposes, the German animal protection act, and the German welfare regulation for laboratory animals. Compliance with aspects of animal welfare law was regularly monitored by the Bayer Animal Health animal welfare commissioner. Cats were kept in cages with adequate floor space and toys for environmental enrichment; cats were generally group-housed by study groups and same gender, while they were kept in individual cages for treatment and fecal sampling on the respective days. The cats were fed with a standard feline diet, and water was provided ad libitum.

Sixteen and 20 purpose-bred, endoparasite-free domestic shorthair cats were enrolled and acclimatized in the respective study facilities, i.e., the Institute for Parasitology, University of Veterinary Medicine Hannover, Germany (Study 1) and Elanco Animal Health, Monheim, Germany (Study 2). Cats enrolled in Study 1 were aged 19–22 weeks and weighed 1.70–2.90 kg, while those enrolled in Study 2 were 26–27 weeks old, weighing 2.40–3.65 kg. A veterinarian examined all cats for study inclusion during the acclimatization period, which was 14 days (Study 1) and 9 days (Study 2), and 1 day before inoculation (both studies).

### Source of *Troglostrongylus brevior* larvae, snail breeding, and infection of snails

For both studies, first-stage larvae (L1) of *T. brevior* were obtained from two naturally infected cats with subclinical troglostrongylosis living in southern (Study 1) and central (Study 2) Italy. Both cats were privately housed, and fecal samples containing L1 were collected after obtaining an informed consent form signed by the owners and required authorizations to perform the activities. Feces were collected daily from the litter box of the two cats from May to November 2019 (Study 1) and from June to August 2020 (Study 2), i.e., the whole duration of the snail infection as described below. Cats were monitored daily for their health and welfare status.

Breeding and management of snails (*Cornu aspersum*) and their infection with L1 and maintenance in vivaria until L3 development were conducted for both studies as previously described for felid metastrongyloids [26]. Snails intended for human consumption were purchased from a farm and divided into two aliquots: 10% were processed by artificial digestion and examined microscopically under a light microscope and subjected to diagnostic polymerase chain reaction (PCR) specific for cat metastrongyloids to verify the absence of natural infections by these parasites [27], and each of the remaining 90% of the snails was infected with 500 L1 of *T. brevior* following procedures described previously [4, 26], then kept in vivaria under controlled conditions of lighting, temperature (approximately 24 °C), and humidity (80%), and fed with vegetables ad libitum.

### Cat allocation and treatment

For both studies, healthy animals that had been acclimatized and met the inclusion criteria (see Table 1) were blocked based on body weight within gender and randomly assigned to one of the two groups, i.e., Group 1 (G1), untreated control, and Group 2 (G2) receiving administration of Advocate^®^ twice at a 4-week interval at the minimum recommended dosage of 0.1 ml/kg body weight. On study day (SD) 0, all cats were anesthetized and experimentally inoculated with *T. brevior* L3 (see “Experimental inoculation of cats” section).

**Table 1:** Animal cohorts, inclusion criteria, and treatment

Activity	Inclusion criterion^a^	Study 1	Study 2
Acclimatization (days)	≥ 7	14^b^	9
Age at first treatment	≥ 10 weeks	19–22 weeks	26–27 weeks
Weight	≥ 1 kg	1.70–2.90 kg	2.40–3.65 kg
Quantitative fecal examination during acclimatization	Negative	Negative	Negative
*N*/Group	N/A	8	10
SD of randomization	N/A	−1	26
SD of inoculation		0	0
SD treatment (Group 2)	N/A	28 and 56	26 and 54

*N/A* not applicable, *SD* study day

^a^Cats were clinically healthy, not pregnant, not excessively fractious, and did not receive any macrocyclic lactone or any drug which could have interfered with the evaluation at least 3 months prior to study start

^b^Due to the extended acclimation period SD −7 activities took place 14 days before inoculation

### Infective dose preparation

Artificial digestion of the snails to obtain infective L3 was performed at SD 0 for both studies based on established protocols [28]. The feet of infected snails were cut with scissors first and then minced with a hand blender. The material was artificially digested for 30 min in a digestion solution (0.6 g pepsin from porcine gastric mucosa [Sigma-Aldrich, P7000, ≥ 2.500 U/mg protein (E1%/280)] and 0.7 ml of 37% HCl in 100 ml of distilled water) at 41 °C on a laboratory shaker. The digested material was filtered using 200 μm and 180 µm sieves in Study 1 and 2, respectively, and centrifuged in 50 ml tubes at 300×*g* for 10 min (Study 1) or at 600×*g* for 5 min (Study 2). The sediment was resuspended in tap water and the centrifugation step was repeated; it was then pooled and shaken, and the larval suspension was set on a magnetic stirrer with a heating plate maintained at 40 °C. While stirring, 10 aliquots of 0.1 ml suspension each were smeared onto glass slides to calculate the mean number of larvae in 0.1 ml. Based on this average, an inoculum volume containing approximately 100 L3 was prepared for the inoculation in both studies.

### Experimental inoculation of cats

Cats of both studies received approximately 100 *T. brevior* L3 on SD 0 as described below. Animals were anesthetized with a combined intramuscular injection of 0.08 ml/kg body weight (BW) Domitor^®^ (1 mg/ml medetomidine HCl, Zoetis) and 0.075 ml/kg BW Ketamin 10%^®^ (100 mg/ml ketamine HCl, WDT). After deep anesthesia, the cat received 0.06 ml/kg BW Emeprid^®^ IM (5 mg/ml, metoclopramide HCl, CEVA) 15 min (Study 1) or a few minutes (Study 2) before inoculation to prevent vomiting or regurgitation. A stomach tube was inserted without (Study 1) or with a rigid endoscope (Study 2). The inoculum was applied via syringe directly into the stomach, the tube was flushed with tap water and pulled out after confirming that no inoculation suspension remained in the tube. All cats were observed for vomiting or regurgitation directly after inoculation for up to 1 h (± 10 min) post-infection.

### Health observations

The health status of cats was observed daily from the start of acclimatization until necropsy in both studies. The detailed schedule of clinical examinations and adverse event observation performed by veterinarians is listed in Table 2.

**Table 2:** Schedule of the clinical examinations and adverse event observation performed in the two efficacy studies

Assessments	Study 1	Study 2
Clinical assessment (health assessment around inoculation and/or treatment time points)	SD 28; SD 29; SD 56; SD 57	SD 0; SD 26; SD 27; SD 54; SD 55
Physical examination (complete clinical examination including auscultation)	SD −7^a^; SD −1	SD −9; SD −1; SD 23; SD 51; SD 63
Respiratory assessment	SD −1; SD 7; SD 14; SD 21; SD 27; SD 35; SD 41; SD 49; SD 55; SD 63; SD 69–72	NA
Adverse event observation	From SD 28 to SD 69/72	From SD 26 to SD 64/65

*NA* not applicable, *SD* study day

^a^Due to the extended acclimation period SD −7 activities took place 14 days before inoculation

### Parasitological examinations

In both studies, individual fecal samples were collected from each cat and examined using quantitative Baermann examination as previously described [29] once daily between SD 18 and 28 (Study 1) and SD 19 and 25 (Study 2) to detect the start of patency. Individual fecal samples were also collected and examined three times per individual cat on SD 35 to 37, SD 63 to 65, and additionally on SDs 42 and 43 (Study 1), or, respectively, three times between SD 48 and 50 and between SD 57 and 63 (Study 2). Larvae were counted and calculated as the number of larvae/g feces (LPG).

### Necropsy

The 16 cats of Study 1 were humanely euthanized on SD 69 to 72 by intravenous application of pentobarbital (0.26 ml/kg BW of Euthadorm^®^ 500 mg/ml, CP Pharma). Cats of Study 2 were humanely euthanized on SD 64 to 65 by intravenous application of pentobarbital (1.25 ml/kg BW of Narcoren^®^, 0.16 g/ml pentobarbital sodium, Boehringer Ingelheim). For each animal, the thorax was opened, and lungs, trachea, and heart were removed completely. The airways and lung tissues were checked for parasites by dissecting piece by piece under a stereomicroscope. All recently dead intact worms were counted as viable worms. Worm fragments were counted only if the anterior end or the posterior end was present. Each anterior and/or posterior end was counted. If the number of anterior ends was greater than the number of posterior ends, the anterior ends were used to calculate the total number of worms and vice versa.

### Efficacy criteria

For both studies, the primary criterion to evaluate the therapeutic efficacy of Advocate^®^ against adult *T. brevior* was the number of viable adult worms counted at necropsy. The efficacy percentage was evaluated based on the geometric mean (GM) according to the recommendations for controlled tests in VICH GL7 (see the % effectiveness formulae below). Appropriate descriptive statistical analysis (number of animals positive for *T. brevior* and GM worm counts per group) was calculated for the parasite burdens of each group. Adequacy of infection was considered met if in ≥ 6 cats of each control group 5 or more adults of *T. brevior* were found.

$$\% {\text{Effectiveness}}\left( {{\text{reduction}}} \right) = \left( {N2 - N1} \right)/N2 \times 100,$$

*N*1 = GM count of *T. brevior* for G2. *N*2 = GM count of *T. brevior* for G1.

## Results

### Inclusion criteria, health observations, and safety assessment

All the 16 and 20 cats met the inclusion criteria and were randomized into treatment groups in the respective studies on SD −1 (Study 1) and SD 26 (Study 2), respectively. Detailed information on the clinical alterations in cats of both studies is listed in Tables 3 and 4.

**Table 3:** Clinical signs observed in cats of study 1

Group	Animal ID	SD	Clinical signs
1 (untreated control)	6331	−7^a^; −1	Underweight
	6535	−7^a^	Slightly underweight
	6577	−1	Abdomen strained
	6598	−7^a^	Slightly underweight
		41; 49	Sniffing
		55	Deepened respiratory sound and sniffing
	6577	70	Deepened respiratory sounds
2 (Advocate^®^)	6366	−3/−2.2^a^	Diarrhea/loose feces
		−1	Pale mucous membranes
	6523	35	Abdomen bloated
	6581	51	Vomiting
	6586	35	Abdomen bloated
	6619	28/29	Mucous to throaty vocalization
		50	Vomiting and diarrhea
		72	Slightly deepened respiratory sounds
	6627	72	Sniffing
	6348, 6523, 6531, 6586, 6366	56	Loose feces with blood in one of the litter boxes^b^

*SD* study day

^a^Due to the extended acclimation period, SD −7 activities took place 14 days before inoculation

^b^Observed in group-housed cats

**Table 4:** Clinical signs observed in cats of study 2

Group	Subject ID	SD	Clinical signs
1 (untreated control)	3263	0	Vomiting
	4011	20	Vomiting, slight diarrhea
		23	Soft/loose stool
	4016	−8	Vomiting
		1	Vomiting
	3261, 3263, 3264	27	Vomited mash/feed found in the box^a^
	4011, 4012	27	Vomited mash/feed found in the box^a^
2 (Advocate^®^)	3262	0	Vomiting
		27	Slight diarrhea/loose feces
	3269	22	Soft/loose feces
		23	Soft/loose feces
	3951	26	Coughing
		27	Vomiting
	3955	21	Vomiting
	3959	0	Vomiting
		27	Coughing

*SD* study day

^a^Observed in group-housed cats

### Parasitological examinations

Between SD 20 and SD 25, all the cats enrolled in both studies started shedding *T. brevior* L1. Overall, 6/8 and 8/10 control cats in Study 1 and 2, respectively, shed L1 up to necropsy, while at the first post-treatment parasitological examination performed on SD 35 (Study 1) and SD 48 (Study 2) the larval shedding ceased in all G2 cats. Detailed information on larval shedding is shown in Tables 5 and 6.

**Table 5:** Fecal larval counts observed pre-treatment and after the first and second treatments in cats included in study 1

Pre-treatment						
	SD 18/19	SD 20	SD 22	SD 24	SD 26	SD 28
No. of cats shedding larvae	0	3	16	16	15	16
Minimum LPG	0	0	4.2	24	0	0.2
Maximum LPG	0	0.6	56	231	492	1896
Arithmetic mean	0.00	0.08	25.63	100.88	154.88	379.14

After first treatment (SD 28)						
	SD 35	SD 36	SD 37	SD 42	SD 43	
Group 1						
No. of cats shedding larvae	8	8	8	7	8	
Minimum LPG	13.8	32.6	9	0	0.4	
Maximum LPG	3255	1011	594	1464	1590	
Arithmetic mean	444.15	251.40	171.78	345.35	490.35	
Group 2						
No. of cats shedding larvae	2	1	0	0	0	
Minimum LPG	0	0	0	0	0	
Maximum LPG	2	1.4	0	0	0	
Arithmetic mean	0.30	0.18	0	0	0	

After second treatment (SD 56)						
	SD 63	SD 64	SD 65			
Group 1						
No. of cats shedding larvae	7	6	6			
Minimum LPG	0	0	0			
Maximum LPG	669.6	3364	2016			
Arithmetic mean	160.88	1170.88	484.50			
Group 2						
No. of cats shedding larvae	0	0	0			
Minimum LPG	0	0	0			
Maximum LPG	0	0	0			
Arithmetic mean	0	0	0			

*SD* study day, *LPG* larvae per gram of feces

**Table 6:** Fecal larval counts observed pre-treatment and after the first and second treatments in cats included in study 2

Pre-treatment						
	SD 19	SD 20	SD 21	SD 22^a^	SD 23^b^	SD 25^b^
No. of cats shedding larvae	0	0	13	18	20	20
Minimum LPG	0	0	0	–	–	–
Maximum LPG	0	0	21	–	–	–
Arithmetic mean	0	0	3.02	2.61^a^	0.2^b^	7.0^b^

After first treatment (SD 26)						
	SD 48	SD 49	SD 50			
Group 1						
No. of cats shedding larvae	10	6	10			
Minimum LPG	1.2	0	0.39			
Maximum LPG	321.57	435.1	4500			
Arithmetic mean	86.35	110.97	741.98			
Group 2						
No. of cats shedding larvae	0	0	0			
Minimum LPG	0	0	0			
Maximum LPG	0	0	0			
Arithmetic mean	0	0	0			

After second treatment (SD 54)						
	SD 57	SD 60	SD 61–63			
Group 1						
No. of cats shedding larvae	9	8	8			
Minimum LPG	0	0	0			
Maximum LPG	594.12	444	926.47			
Arithmetic mean	152.60	125.93	279.83			
Group 2						
No. of cats shedding larvae	0	0	0			
Minimum LPG	0	0	0			
Maximum LPG	0	0	0			
Arithmetic mean	0	0	0			

*SD* study day, *LPG* larvae per gram of feces

^a^Only samples from 7 cats that were not shedding larvae on SD 21 were collected

^b^Only samples from 2 cats that were not shedding larvae on SD 22 were collected

### Pathological findings

Gross pathological findings were recorded for the cats of Study 2. Eight out of 10 of the untreated cats (G1) showed areas of meat-like consistency and bright color with purulent mucus in the bronchi, while the remaining two cats did not show such lesions. In the group of treated cats (G2), marbled and inhomogeneous pulmonary tissue was observed in one cat and the presence of a 0.2 × 0.2 mm small, rough nodule was observed in another cat, while the remaining eight cats did not show any pulmonary lesions.

### Adult worm count

*Troglostrongylus brevior* adult worms were found in 4/8 and 4/10 untreated (G1) animals in Study 1 and 2, respectively. All worms were either viable or recently dead and intact. In two of these cats living larvae and eggs of *T. brevior* were also found. No adult *T. brevior* worms or other development stages were detected in any of the cats treated with Advocate^®^ (Table 7). The worm counts in control cats did not meet relevant GL requirements for adequacy of infection (i.e., a minimum of 6 cats of the control groups with adult worms detected at necropsy), thus statistical analysis was not performed.

**Table 7:** *Troglostrongylus brevior* worm counts at necropsy in cats untreated and treated with Advocate^®^

	Study group	Treatment	Cats with lungworms/number of worms	Cats without detectable lungworms	Worm counts (geometric mean)	%
Study 1	G1	Untreated	4/10–42	4	3.82	NA
	G2	Advocate^®^	0	8	0	100^a^
Study 2	G1	Untreated	4/16–28	6	2.12	NA
	G2	Advocate^®^	0	10	0	100^a^

*NA* not applicable

^a^Not proven statistically due to a lack of adequate infection in the control group

## Discussion

*Troglostrongylus brevior* is an emerging nematode which may cause severe parasitic bronchopneumonia. The disease can be life-threatening especially for kittens and young animals and permanent damages, such as irreversible pulmonary hypertension and chronic complications, may occur [1]. Subclinical infections are also of importance [22, 30], as undiagnosed cats represent a source of infection for intermediate hosts. Effective treatment is thus critically important to cure infected cats with respiratory signs and to stop larval shedding and interrupt the life cycle of *T. brevior*.

Very few options are available to date for treating cat troglostrongylosis, and only two spot-on formulations containing eprinomectin (Broadline™ and Nexgard^®^ Combo, Boehringer Ingelheim) are licensed in the EU market to treat *T. brevior* infections [18–20]. Other molecules have also been proven potentially efficacious in terms of larval shedding and complete clinical recovery in cats infected with *T. brevior* either in monospecific or in mixed infection with *A. abstrusus*. This is the case of the macrocyclic lactone milbemycin oxime in some clinical cases [16], and of the cyclooctadepsipeptide emodepside (in combination with praziquantel) in a purposed field trial which evaluated the efficacy of two administrations 2 weeks apart under natural conditions [21]. The efficacy of oral fenbendazole against *T. brevior* has been suggested [31] but has never been evaluated or demonstrated [1].

The spot-on formulation containing 1% moxidectin (Advocate^®^) here investigated is already labelled for treating and preventing the infection caused by *A. abstrusus* [1]. In recent years its potential usefulness against *T. brevior* was preliminarily shown in case reports and clinical studies [15, 16], and a more recent field trial proved 100% effectiveness in stopping *T. brevior* L1 shedding in cats with subclinical natural infections [22]. The efficacy of topical moxidectin in stopping *T. brevior* larval shedding was also demonstrated in a study evaluating the larval infectivity in mollusc intermediate hosts [32] and in a clinical case of a wildcat with multiple respiratory infections including troglostrongylosis [7].

Overall, the results obtained from these two studies show that moxidectin administered topically at the dose of 1 mg/kg BW is efficacious and safe in treating *T. brevior* infections under experimental conditions, further corroborating the preliminary evidence obtained in naturally infected cats [22]. The efficacy is convincing albeit some unexpected limitations occurred in the two studies. No statistical analyses were performed because the VICH GL prerequisite, i.e., adequacy of infection in control cats (i.e., six cats with detectable worms upon necropsy), was not met. However, the experimental infection was successful in both studies as all cats were shedding larvae within the previously reported prepatent period of approximately 3–4 weeks [17, 19], i.e., by SD 22 (Study 1) and 23 (Study 2), respectively.

The lack of adult worms in some untreated cats despite larval shedding in the days before/at necropsy can be explained by a possible reduced worm life span and spontaneous death of the parasites due to immune mechanisms. Although unlikely considering the thorough examination of the lungs, it cannot be ultimately ruled out that the presence of a few worms located deep in the airways could have impaired their retrieval at necropsy. Field studies have shown that the rate of occurrence of *T. brevior* decreases with the age of animals, as it is more frequent in animals aged less than 6 months, less diagnosed in 6–24-month-old cats, and seldom or not detected in cats older than 2 years [9, 33]. As the cats of Study 2 were ≥ 6.5 months old when the patent infection was established, it can be therefore assumed that in some animals, anatomical and immunity factors (e.g., inflammatory mediators) induced a spontaneous elimination of adult worms. Accordingly, the percentage of cats with adult *T. brevior* worms in Study 1 (aged 4.7–5.5 months) was higher though some were still negative for adult parasites. Although some control cats were negative for adult *T. brevior* worms at necropsy, in both studies no larval shedding was detected in cats of G2 after treatment while most control cats (6/8 and 8/10 in Study 1 and 2, respectively) continued to shed L1 until the end of the study. Given that all control cats shed L1 until SD 43 and 50 in Study 1 and 2 respectively, it can be argued that adult worms were present in these cats during later phases of the study, well beyond the time point larval shedding had ceased in G2 animals, and that adult parasites began to die, naturally and/or for cat immunity response, between 6 and 9 weeks post-infection.

One cat of Study 1 showed respiratory signs on SD 28/29 starting from 4 h after the first treatment. Similarly, two cats of Study 2 displayed respiratory clinical signs 4 h and 24 h after the first treatment respectively. These findings overlap those of a recent field trial investigating the efficacy of emodepside in treating feline troglostrongylosis where two cats showed a temporary worsening of their clinical status possibly related to an inflammatory response to the death of nematodes [21]. The different number of cats displaying respiratory signs, i.e., five cats in Study 1 versus two cats in Study 2, can be explained by the age of the animals when they were experimentally inoculated, i.e., 19/22 weeks for Study 1 and 26/27 weeks for Study 2, respectively. In fact, the age of the animals has an influence on the clinical severity as well as the infection rates, i.e., the most relevant clinical pictures occur in cats ≤ 6 months and in kittens that acquire the infection vertically [1, 3, 16, 34]. The persistence of the clinical signs after the second administration of Advocate^®^ on SD 72 in two cats of G2 of Study 1 is probably due to the high pathogenic potential of *T. brevior* in kittens, in which it can induce long-term consequences despite the administration of appropriate treatment [15].

The few adverse events detected in both studies were unlikely related to the administration of Advocate^®^. Hence, it can be stated that the treatment was well tolerated in all study cats and confirms the safety data already obtained in natural conditions [22].

## Conclusions

The results of the present in vivo studies under experimental conditions confirm the efficacy and safety of Advocate^®^ in the treatment of feline troglostrongylosis, as already shown under natural conditions in single reports and cases series, and in a purpose field trial [15, 16, 22]. Therefore, Advocate^®^ can be considered a suitable choice for the treatment of cat troglostrongylosis even after a single administration. Given that it has been shown that moxidectin contained in different products is efficacious for the prevention of aelurostrongylosis [35, 36], its ability in the prevention of troglostrongylosis is highly worthy of further investigation.

## Data Availability

All the data generated, and methodology applied in the present study supporting reported results are included in the manuscript.

## Abbreviations

*A.*: *Aelurostrongylus*
BW: Body weight
EU: European Union
*F*.: *Felis*
G: Group
GL: Guideline
GM: Geometric mean
HCl: Hydrochloric acid
L: Larva(e)
LPG: Larvae per gram
PCR: Polymerase chain reaction
SD: Study day
*T*.: *Troglostrongylus*
VICH: International Cooperation on Harmonisation of Technical Requirements for Registration of Veterinary Medicinal Products
w/v: Weight per volume
